# 2-Amino-5-chloro­pyridinium trifluoro­acetate

**DOI:** 10.1107/S1600536810008196

**Published:** 2010-03-10

**Authors:** Madhukar Hemamalini, Hoong-Kun Fun

**Affiliations:** a X-ray Crystallography Unit, School of Physics, Universiti Sains Malaysia, 11800 USM, Penang, Malaysia

## Abstract

The asymmetric unit of the title salt, C_5_H_6_ClN_2_^+^·C_2_F_3_O_2_^−^, contains two independent 2-amino-5-chloro­pyridinium cations and two independent trifluoro­acetate anions. The F atoms of both anions are disordered over two sets of positions, with occupancy ratios of 0.672 (12):0.328 (12) and 0.587 (15):0.413 (15). In the crystal, the cations and anions are linked *via* N—H⋯O and C—H⋯O hydrogen bonds, forming a two-dimensional network parallel to (001).

## Related literature

For background to the chemistry of substituted pyridines, see: Pozharski *et al.* (1997 ▶); Katritzky *et al.* (1996 ▶). For related structures, see: Pourayoubi *et al.* (2007 ▶); Hemamalini & Fun (2010*a* ▶,*b* ▶,*c* ▶). For details of hydrogen bonding, see: Jeffrey & Saenger (1991 ▶); Jeffrey (1997 ▶); Scheiner (1997 ▶). For hydrogen-bond motifs, see: Bernstein *et al.* (1995 ▶). For bond-length data, see: Allen *et al.* (1987 ▶).
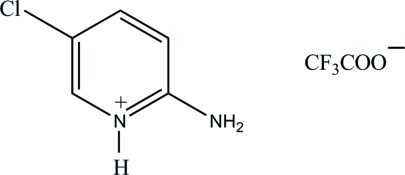


## Experimental

### 

#### Crystal data

C_5_H_6_ClN_2_^+^·C_2_F_3_O_2_^−^
*M* *_r_* = 242.59
Monoclinic, 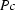

*a* = 5.0377 (1) Å
*b* = 11.2923 (2) Å
*c* = 17.5386 (3) Å
β = 90.001 (1)°
*V* = 997.72 (3) Å^3^
*Z* = 4
Mo *K*α radiation
μ = 0.41 mm^−1^
*T* = 296 K
0.43 × 0.26 × 0.14 mm

#### Data collection

Bruker SMART APEXII CCD area-detector diffractometer
Absorption correction: multi-scan (*SADABS*; Bruker, 2009 ▶) *T*_min_ = 0.842, *T*_max_ = 0.945
17652 measured reflections
4388 independent reflections
3191 reflections with *I* > 2σ(*I*)
*R*_int_ = 0.027

#### Refinement

*R*[*F* ^2^ > 2σ(*F* ^2^)] = 0.036
*wR*(*F* ^2^) = 0.094
*S* = 1.03
4388 reflections
375 parameters
110 restraints
H atoms treated by a mixture of independent and constrained refinement
Δρ_max_ = 0.12 e Å^−3^
Δρ_min_ = −0.15 e Å^−3^
Absolute structure: Flack (1983 ▶), 2096 Friedel pairs
Flack parameter: 0.01 (7)

### 

Data collection: *APEX2* (Bruker, 2009 ▶); cell refinement: *SAINT* (Bruker, 2009 ▶); data reduction: *SAINT*; program(s) used to solve structure: *SHELXTL* (Sheldrick, 2008 ▶); program(s) used to refine structure: *SHELXTL*; molecular graphics: *SHELXTL*; software used to prepare material for publication: *SHELXTL* and *PLATON* (Spek, 2009 ▶).

## Supplementary Material

Crystal structure: contains datablocks global, I. DOI: 10.1107/S1600536810008196/ci5043sup1.cif

Structure factors: contains datablocks I. DOI: 10.1107/S1600536810008196/ci5043Isup2.hkl

Additional supplementary materials:  crystallographic information; 3D view; checkCIF report

## Figures and Tables

**Table 1:** Hydrogen-bond geometry (Å, °)

*D*—H⋯*A*	*D*—H	H⋯*A*	*D*⋯*A*	*D*—H⋯*A*
N1*A*—H1*NA*⋯O1*A*^i^	0.94 (3)	1.79 (3)	2.727 (3)	173 (3)
N2*A*—H2*NA*⋯O2*A*^i^	0.90 (3)	1.95 (3)	2.840 (4)	175 (3)
N2*A*—H3*NA*⋯O1*B*^ii^	0.87 (3)	2.00 (2)	2.863 (3)	171 (4)
N1*B*—H1*NB*⋯O1*B*^iii^	0.87 (3)	1.87 (3)	2.734 (3)	175 (3)
N2*B*—H2*NB*⋯O2*B*^iii^	0.90 (2)	1.94 (2)	2.838 (4)	170 (2)
N2*B*—H3*NB*⋯O1*A*	0.87 (3)	1.99 (2)	2.861 (3)	175 (4)
C5*A*—H5*AA*⋯O2*B*^i^	0.97 (3)	2.29 (3)	3.210 (4)	158 (3)
C5*B*—H5*BA*⋯O2*A*^iv^	0.96 (3)	2.31 (3)	3.208 (3)	157 (3)

Symmetry codes: (i) 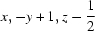; (ii) 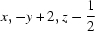; (iii) 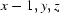; (iv) 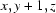.

**Fig. 1.**
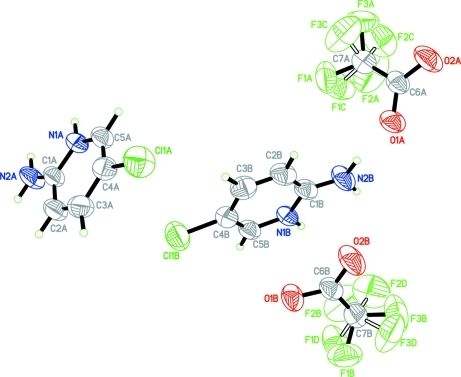
The asymmetric unit of the title compound. Displacement ellipsoids are drawn at the 50% probability level. All disorder components are shown.

**Fig. 2.**
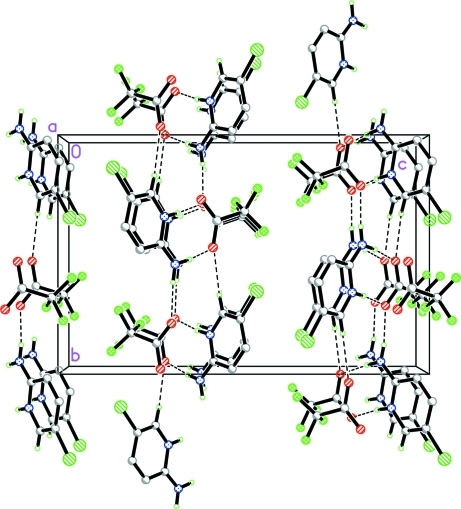
The crystal packing of the title compound, showing the hydrogen-bonded (dashed lines) networks.

## supplementary crystallographic information

### Comment

Pyridine and its derivatives play an important role in heterocyclic chemistry (Pozharski *et al.*, 1997; Katritzky *et al.*, 1996). They are often involved in hydrogen-bond interactions (Jeffrey & Saenger, 1991; Jeffrey, 1997; Scheiner, 1997). We have recently reported the crystal structures of 2-amino-5-chloropyridinium 4-hydroxybenzoate (Hemamalini & Fun, 2010a), 2-amino-5-chloropyridine benzoic acid (Hemamalini & Fun, 2010b) and 2-amino-5-chloropyridinium hydrogen succinate. (Hemamalini & Fun, 2010c). In continuation of our studies of pyridinium derivatives, the crystal structure determination of the title compound has been undertaken.

The asymmetric unit of the title compound consists of two crystallographically independent 2-amino-5-chloropyridinium cations (A and B) and two trifluoroacetate anions (A and B) (Fig. 1).  Each 2-amino-5-chloropyridinium cation is planar, with a maximum deviation of 0.017 (3) Å for atom C3A in cation A and 0.026 (1) Å for atom C1B in cation B. In the cations, protonation at atoms N1A and N1B lead to a slight increase in the C1A–N1A–C5A [122.7 (3)°] and C1B—N1B—C5B [123.2 (3)°] angles compared to those observed in an unprotonated structure (Pourayoubi *et al.*, 2007). Bond lengths and angles are normal (Allen *et al.*, 1987).

In the crystal packing (Fig. 2), the A/B type 2-amino-5-chloropyridinium cations interact with the carboxylate groups of the A/B type trifluoroacetate anions through a pair of N—H···O  hydrogen bonds, forming an *R*_2_^2^(8) (Bernstein *et al.*, 1995) ring motif.  The packing is further stabilized by weak C5A—H5AA···O2B and C5B—H5BA···O2A (Table 1) hydrogen bonds.

### Experimental

To a hot methanol solution (20 ml) of 2-amino-5-chloropyridine (27 mg, Aldrich) was added a few drops of trifluoroacetic acid.  The solution was warmed over a water bath for a few minutes. The resulting solution was allowed to cool slowly to room temperature. Crystals of the title compound appeared after a few days.

### Refinement

All H atoms were located in a difference Fourier map and refined [N—H =0.87 (2)–0.94 (3) Å and C—H =0.94 (4)–0.98 (4) Å]; the N–H distances of the NH_2_ groups were restrained to be equal. The F atoms of both anions are disordered over two positions, with site occupancies of 0.672 (12) and 0.328 (12) in one of the anions, and 0.587 (15):0.413 (15) in the other anion. In each anion, the C—F distances were restrained to be equal and the U^ij^ components of F atoms were restrained to an approximate isotropic behaviour.

### Crystal data

**Table:**

C_5_H_6_ClN_2_^+^·C_2_F_3_O_2_^−^	*F*(000) = 488
*M**_r_* = 242.59	*D*_x_ = 1.615 Mg m^−^^3^
Monoclinic, *P**c*	Mo *K*α radiation, λ = 0.71073 Å
Hall symbol: P -2yc	Cell parameters from 6764 reflections
*a* = 5.0377 (1) Å	θ = 2.9–23.0°
*b* = 11.2923 (2) Å	µ = 0.41 mm^−^^1^
*c* = 17.5386 (3) Å	*T* = 296 K
β = 90.001 (1)°	Blcok, colourless
*V* = 997.72 (3) Å^3^	0.43 × 0.26 × 0.14 mm
*Z* = 4	

### Data collection

**Table:**

Bruker SMART APEXII CCD area-detector diffractometer	4388 independent reflections
Radiation source: fine-focus sealed tube	3191 reflections with *I* > 2σ(*I*)
graphite	*R*_int_ = 0.027
φ and ω scans	θ_max_ = 27.5°, θ_min_ = 1.8°
Absorption correction: multi-scan (*SADABS*; Bruker, 2009)	*h* = −6→6
*T*_min_ = 0.842, *T*_max_ = 0.945	*k* = −14→14
17652 measured reflections	*l* = −22→22

### Refinement

**Table:**

Refinement on *F*^2^	Secondary atom site location: difference Fourier map
Least-squares matrix: full	Hydrogen site location: inferred from neighbouring sites
*R*[*F*^2^ > 2σ(*F*^2^)] = 0.036	H atoms treated by a mixture of independent and constrained refinement
*wR*(*F*^2^) = 0.094	*w* = 1/[σ^2^(*F*_o_^2^) + (0.0449*P*)^2^ + 0.0781*P*] where *P* = (*F*_o_^2^ + 2*F*_c_^2^)/3
*S* = 1.03	(Δ/σ)_max_ = 0.001
4388 reflections	Δρ_max_ = 0.12 e Å^−^^3^
375 parameters	Δρ_min_ = −0.15 e Å^−^^3^
110 restraints	Absolute structure: Flack (1983), 2096 Friedel pairs
Primary atom site location: structure-invariant direct methods	Flack parameter: 0.01 (7)

### Special details

**Table:**

Geometry. All s.u.'s (except the s.u. in the dihedral angle between two l.s. planes) are estimated using the full covariance matrix. The cell s.u.'s are taken into account individually in the estimation of s.u.'s in distances, angles and torsion angles; correlations between s.u.'s in cell parameters are only used when they are defined by crystal symmetry. An approximate (isotropic) treatment of cell s.u.'s is used for estimating s.u.'s involving l.s. planes.
Refinement. Refinement of F^2^ against ALL reflections. The weighted R-factor wR and goodness of fit S are based on F^2^, conventional R-factors R are based on F, with F set to zero for negative F^2^. The threshold expression of F^2^ > 2σ(F^2^) is used only for calculating R-factors(gt) etc. and is not relevant to the choice of reflections for refinement. R-factors based on F^2^ are statistically about twice as large as those based on F, and R- factors based on ALL data will be even larger.

### Fractional atomic coordinates and isotropic or equivalent isotropic displacement parameters (Å^2^)

**Table:**

	*x*	*y*	*z*	*U*_iso_*/*U*_eq_	Occ. (<1)
Cl1A	1.1299 (2)	0.65347 (8)	0.53508 (6)	0.0863 (3)	
N1A	0.6452 (5)	0.84270 (19)	0.40858 (15)	0.0509 (6)	
N2A	0.5936 (6)	1.0402 (2)	0.38078 (18)	0.0688 (7)	
C1A	0.7219 (6)	0.9568 (2)	0.41821 (16)	0.0535 (7)	
C2A	0.9320 (6)	0.9791 (3)	0.46873 (17)	0.0620 (7)	
C3A	1.0548 (7)	0.8880 (3)	0.50499 (18)	0.0657 (8)	
C4A	0.9711 (6)	0.7707 (2)	0.49139 (16)	0.0598 (7)	
C5A	0.7678 (6)	0.7505 (3)	0.44427 (18)	0.0547 (7)	
Cl1B	0.6300 (2)	0.84657 (8)	0.66202 (6)	0.0862 (3)	
N1B	0.1456 (5)	0.65696 (18)	0.78845 (16)	0.0515 (6)	
N2B	0.0936 (6)	0.4597 (2)	0.81654 (19)	0.0696 (7)	
C1B	0.2208 (6)	0.5431 (2)	0.77885 (16)	0.0536 (7)	
C2B	0.4312 (6)	0.5216 (3)	0.72826 (17)	0.0622 (7)	
C3B	0.5552 (7)	0.6116 (3)	0.69219 (18)	0.0647 (8)	
C4B	0.4720 (6)	0.7291 (2)	0.70553 (16)	0.0592 (7)	
C5B	0.2675 (6)	0.7496 (2)	0.75301 (17)	0.0543 (7)	
F1A	0.1120 (15)	0.2688 (5)	0.6659 (3)	0.103 (2)	0.672 (12)
F2A	−0.2319 (10)	0.2039 (11)	0.7180 (3)	0.145 (3)	0.672 (12)
F3A	−0.002 (2)	0.0953 (5)	0.6438 (3)	0.133 (3)	0.672 (12)
F1C	−0.050 (4)	0.2829 (7)	0.6917 (10)	0.120 (5)	0.328 (12)
F2C	−0.217 (2)	0.1179 (12)	0.6924 (8)	0.113 (4)	0.328 (12)
F3C	0.135 (3)	0.1402 (18)	0.6367 (6)	0.143 (6)	0.328 (12)
O1A	0.2546 (5)	0.21708 (17)	0.80805 (12)	0.0645 (5)	
O2A	0.1855 (6)	0.0286 (2)	0.77827 (16)	0.0847 (7)	
C6A	0.1664 (6)	0.1353 (3)	0.76755 (19)	0.0562 (7)	
C7A	0.0082 (7)	0.1732 (3)	0.69751 (19)	0.0725 (9)	
F1B	0.6266 (18)	0.7635 (7)	1.0334 (4)	0.106 (2)	0.587 (15)
F2B	0.2762 (14)	0.7160 (13)	0.9780 (4)	0.134 (3)	0.587 (15)
F3B	0.473 (3)	0.5940 (5)	1.0508 (5)	0.129 (3)	0.587 (15)
F1D	0.490 (4)	0.7839 (5)	1.0133 (8)	0.120 (4)	0.413 (15)
F2D	0.2692 (17)	0.6301 (14)	0.9986 (7)	0.123 (4)	0.413 (15)
F3D	0.615 (3)	0.6227 (14)	1.0603 (5)	0.134 (4)	0.413 (15)
O1B	0.7544 (5)	0.71703 (17)	0.88936 (12)	0.0642 (5)	
O2B	0.6855 (6)	0.5286 (2)	0.91889 (15)	0.0840 (7)	
C6B	0.6665 (6)	0.6354 (3)	0.92942 (19)	0.0560 (7)	
C7B	0.5093 (7)	0.6735 (3)	0.99991 (19)	0.0718 (9)	
H1NA	0.507 (7)	0.829 (3)	0.3734 (17)	0.059 (8)*	
H2NA	0.470 (6)	1.021 (3)	0.3463 (17)	0.073 (10)*	
H3NA	0.652 (7)	1.112 (2)	0.388 (2)	0.071 (10)*	
H2AA	0.998 (8)	1.056 (4)	0.477 (2)	0.081 (10)*	
H3AA	1.194 (7)	0.905 (3)	0.543 (2)	0.073 (10)*	
H5AA	0.694 (6)	0.673 (3)	0.4342 (16)	0.050 (7)*	
H1NB	0.023 (7)	0.672 (3)	0.8221 (18)	0.059 (9)*	
H2NB	−0.023 (5)	0.479 (2)	0.8537 (14)	0.057 (8)*	
H3NB	0.143 (8)	0.386 (2)	0.811 (2)	0.079 (11)*	
H2BA	0.494 (7)	0.448 (3)	0.719 (2)	0.077 (10)*	
H3BA	0.701 (8)	0.598 (4)	0.656 (2)	0.081 (11)*	
H5BA	0.192 (7)	0.826 (3)	0.7621 (19)	0.065 (9)*	

### Atomic displacement parameters (Å^2^)

**Table:**

	*U*^11^	*U*^22^	*U*^33^	*U*^12^	*U*^13^	*U*^23^
Cl1A	0.0909 (6)	0.0709 (6)	0.0971 (6)	0.0149 (5)	−0.0160 (5)	0.0077 (5)
N1A	0.0552 (15)	0.0349 (13)	0.0625 (15)	−0.0015 (9)	−0.0004 (12)	−0.0045 (10)
N2A	0.082 (2)	0.0340 (13)	0.0903 (19)	−0.0087 (13)	−0.0106 (16)	0.0013 (13)
C1A	0.0606 (17)	0.0382 (15)	0.0618 (17)	−0.0065 (12)	0.0072 (14)	−0.0057 (12)
C2A	0.0690 (19)	0.0444 (15)	0.0727 (18)	−0.0111 (14)	0.0036 (15)	−0.0073 (13)
C3A	0.065 (2)	0.067 (2)	0.0655 (19)	−0.0096 (16)	−0.0006 (16)	−0.0126 (15)
C4A	0.0682 (19)	0.0514 (15)	0.0597 (15)	0.0034 (14)	0.0031 (14)	−0.0035 (12)
C5A	0.0636 (19)	0.0372 (14)	0.0634 (16)	−0.0017 (13)	0.0059 (14)	−0.0033 (12)
Cl1B	0.0915 (6)	0.0693 (6)	0.0976 (6)	−0.0155 (5)	0.0171 (5)	0.0067 (5)
N1B	0.0584 (16)	0.0329 (13)	0.0632 (16)	0.0015 (10)	0.0008 (12)	−0.0044 (10)
N2B	0.084 (2)	0.0342 (13)	0.0906 (19)	0.0067 (13)	0.0139 (16)	0.0000 (13)
C1B	0.0582 (17)	0.0381 (16)	0.0644 (18)	0.0067 (12)	−0.0075 (14)	−0.0054 (12)
C2B	0.0684 (19)	0.0443 (15)	0.0737 (18)	0.0119 (14)	−0.0043 (15)	−0.0100 (13)
C3B	0.067 (2)	0.0648 (19)	0.0627 (18)	0.0089 (16)	0.0010 (16)	−0.0096 (15)
C4B	0.0669 (18)	0.0516 (15)	0.0592 (15)	−0.0050 (14)	−0.0034 (14)	−0.0023 (12)
C5B	0.0641 (19)	0.0370 (14)	0.0617 (16)	0.0009 (13)	−0.0068 (14)	−0.0045 (12)
F1A	0.137 (5)	0.087 (3)	0.085 (3)	−0.028 (3)	−0.016 (2)	0.036 (2)
F2A	0.079 (3)	0.232 (8)	0.126 (4)	0.045 (4)	−0.002 (2)	0.037 (5)
F3A	0.211 (7)	0.091 (3)	0.096 (3)	−0.004 (3)	−0.049 (4)	−0.036 (2)
F1C	0.159 (10)	0.061 (4)	0.141 (8)	0.016 (6)	−0.064 (7)	−0.002 (5)
F2C	0.088 (6)	0.104 (7)	0.147 (8)	−0.016 (5)	−0.041 (5)	0.011 (6)
F3C	0.148 (9)	0.204 (11)	0.078 (6)	−0.006 (7)	0.005 (6)	−0.007 (7)
O1A	0.0824 (15)	0.0373 (11)	0.0737 (13)	0.0111 (9)	−0.0104 (11)	−0.0062 (9)
O2A	0.1066 (19)	0.0374 (13)	0.110 (2)	0.0061 (12)	−0.0211 (15)	−0.0014 (12)
C6A	0.0601 (18)	0.0410 (16)	0.0676 (18)	0.0045 (13)	0.0056 (13)	−0.0019 (13)
C7A	0.089 (3)	0.0580 (19)	0.071 (2)	−0.0068 (18)	−0.0022 (18)	−0.0009 (15)
F1B	0.122 (5)	0.110 (5)	0.085 (3)	−0.021 (3)	0.008 (3)	−0.041 (3)
F2B	0.086 (4)	0.196 (8)	0.122 (4)	0.049 (5)	0.006 (3)	−0.022 (5)
F3B	0.188 (8)	0.085 (3)	0.116 (4)	−0.012 (4)	0.061 (5)	0.028 (3)
F1D	0.174 (9)	0.052 (3)	0.134 (7)	0.006 (5)	0.077 (7)	−0.007 (4)
F2D	0.072 (4)	0.141 (8)	0.156 (7)	−0.012 (5)	0.030 (4)	−0.026 (6)
F3D	0.150 (8)	0.184 (9)	0.066 (4)	−0.001 (6)	0.001 (5)	0.033 (5)
O1B	0.0833 (15)	0.0377 (11)	0.0715 (13)	0.0099 (9)	0.0124 (11)	0.0067 (9)
O2B	0.1067 (19)	0.0370 (12)	0.1083 (19)	0.0034 (12)	0.0226 (14)	0.0008 (12)
C6B	0.0605 (18)	0.0381 (16)	0.0696 (18)	0.0039 (13)	−0.0061 (13)	0.0001 (13)
C7B	0.089 (3)	0.0571 (19)	0.070 (2)	−0.0047 (18)	0.0054 (18)	0.0018 (15)

### Geometric parameters (Å, °)

**Table:**

Cl1A—C4A	1.726 (3)	C2B—H2BA	0.90 (4)
N1A—C1A	1.355 (4)	C3B—C4B	1.411 (5)
N1A—C5A	1.362 (4)	C3B—H3BA	0.98 (4)
N1A—H1NA	0.94 (3)	C4B—C5B	1.345 (4)
N2A—C1A	1.317 (4)	C5B—H5BA	0.95 (4)
N2A—H2NA	0.90 (2)	F1A—C7A	1.321 (4)
N2A—H3NA	0.87 (2)	F2A—C7A	1.308 (5)
C1A—C2A	1.403 (4)	F3A—C7A	1.290 (5)
C2A—C3A	1.358 (5)	F1C—C7A	1.276 (7)
C2A—H2AA	0.94 (4)	F2C—C7A	1.299 (7)
C3A—C4A	1.411 (5)	F3C—C7A	1.299 (7)
C3A—H3AA	0.98 (4)	O1A—C6A	1.247 (4)
C4A—C5A	1.336 (4)	O2A—C6A	1.223 (4)
C5A—H5AA	0.97 (3)	C6A—C7A	1.525 (5)
Cl1B—C4B	1.725 (3)	F1B—C7B	1.314 (5)
N1B—C1B	1.351 (4)	F2B—C7B	1.325 (5)
N1B—C5B	1.363 (4)	F3B—C7B	1.280 (5)
N1B—H1NB	0.87 (3)	F1D—C7B	1.272 (6)
N2B—C1B	1.317 (4)	F2D—C7B	1.306 (6)
N2B—H2NB	0.902 (19)	F3D—C7B	1.317 (7)
N2B—H3NB	0.87 (2)	O1B—C6B	1.240 (4)
C1B—C2B	1.404 (4)	O2B—C6B	1.224 (4)
C2B—C3B	1.351 (5)	C6B—C7B	1.530 (5)
			
C1A—N1A—C5A	122.7 (3)	C2B—C3B—C4B	119.5 (3)
C1A—N1A—H1NA	116.9 (18)	C2B—C3B—H3BA	122 (3)
C5A—N1A—H1NA	120.4 (18)	C4B—C3B—H3BA	119 (3)
C1A—N2A—H2NA	120 (2)	C5B—C4B—C3B	119.5 (3)
C1A—N2A—H3NA	115 (3)	C5B—C4B—Cl1B	119.7 (2)
H2NA—N2A—H3NA	124 (3)	C3B—C4B—Cl1B	120.8 (3)
N2A—C1A—N1A	118.5 (3)	C4B—C5B—N1B	119.7 (3)
N2A—C1A—C2A	123.8 (3)	C4B—C5B—H5BA	124 (2)
N1A—C1A—C2A	117.7 (3)	N1B—C5B—H5BA	116 (2)
C3A—C2A—C1A	120.2 (3)	O2A—C6A—O1A	127.9 (3)
C3A—C2A—H2AA	118 (2)	O2A—C6A—C7A	116.2 (3)
C1A—C2A—H2AA	122 (2)	O1A—C6A—C7A	115.9 (3)
C2A—C3A—C4A	119.7 (3)	F1C—C7A—F3C	109.0 (10)
C2A—C3A—H3AA	120 (2)	F1C—C7A—F2C	105.1 (8)
C4A—C3A—H3AA	121 (2)	F3C—C7A—F2C	103.6 (9)
C5A—C4A—C3A	119.6 (3)	F3A—C7A—F2A	110.2 (6)
C5A—C4A—Cl1A	119.9 (2)	F3A—C7A—F1A	105.4 (5)
C3A—C4A—Cl1A	120.4 (3)	F2A—C7A—F1A	105.4 (5)
C4A—C5A—N1A	120.1 (3)	F3A—C7A—C6A	114.6 (4)
C4A—C5A—H5AA	124.3 (18)	F2A—C7A—C6A	109.6 (3)
N1A—C5A—H5AA	115.6 (18)	F1A—C7A—C6A	111.1 (3)
C1B—N1B—C5B	123.2 (3)	O2B—C6B—O1B	128.2 (3)
C1B—N1B—H1NB	118 (2)	O2B—C6B—C7B	116.1 (3)
C5B—N1B—H1NB	118 (2)	O1B—C6B—C7B	115.7 (3)
C1B—N2B—H2NB	120.5 (18)	F1D—C7B—F2D	107.5 (7)
C1B—N2B—H3NB	119 (3)	F3B—C7B—F1B	107.1 (6)
H2NB—N2B—H3NB	119 (3)	F1D—C7B—F3D	108.0 (9)
N2B—C1B—N1B	118.8 (3)	F2D—C7B—F3D	103.0 (7)
N2B—C1B—C2B	124.1 (3)	F3B—C7B—F2B	109.3 (6)
N1B—C1B—C2B	117.1 (3)	F1B—C7B—F2B	104.3 (5)
C3B—C2B—C1B	121.0 (3)	F3B—C7B—C6B	116.1 (4)
C3B—C2B—H2BA	117 (2)	F1B—C7B—C6B	110.3 (4)
C1B—C2B—H2BA	122 (2)	F2B—C7B—C6B	109.0 (4)
			
C5A—N1A—C1A—N2A	179.3 (3)	O2A—C6A—C7A—F3A	−24.8 (7)
C5A—N1A—C1A—C2A	−1.7 (4)	O1A—C6A—C7A—F3A	157.0 (6)
N2A—C1A—C2A—C3A	−179.8 (3)	O2A—C6A—C7A—F3C	−66.6 (11)
N1A—C1A—C2A—C3A	1.2 (4)	O1A—C6A—C7A—F3C	115.3 (11)
C1A—C2A—C3A—C4A	0.3 (5)	O2A—C6A—C7A—F2C	47.5 (10)
C2A—C3A—C4A—C5A	−1.5 (5)	O1A—C6A—C7A—F2C	−130.6 (9)
C2A—C3A—C4A—Cl1A	178.4 (2)	O2A—C6A—C7A—F2A	99.7 (7)
C3A—C4A—C5A—N1A	1.1 (4)	O1A—C6A—C7A—F2A	−78.4 (7)
Cl1A—C4A—C5A—N1A	−178.8 (2)	O2A—C6A—C7A—F1A	−144.2 (5)
C1A—N1A—C5A—C4A	0.5 (4)	O1A—C6A—C7A—F1A	37.6 (5)
C5B—N1B—C1B—N2B	178.9 (3)	O2B—C6B—C7B—F1D	178.7 (12)
C5B—N1B—C1B—C2B	−1.5 (4)	O1B—C6B—C7B—F1D	−2.6 (12)
N2B—C1B—C2B—C3B	−179.1 (3)	O2B—C6B—C7B—F3B	17.6 (8)
N1B—C1B—C2B—C3B	1.3 (4)	O1B—C6B—C7B—F3B	−163.7 (8)
C1B—C2B—C3B—C4B	0.0 (5)	O2B—C6B—C7B—F2D	−56.8 (10)
C2B—C3B—C4B—C5B	−1.1 (5)	O1B—C6B—C7B—F2D	121.9 (9)
C2B—C3B—C4B—Cl1B	178.7 (3)	O2B—C6B—C7B—F1B	139.6 (6)
C3B—C4B—C5B—N1B	0.9 (4)	O1B—C6B—C7B—F1B	−41.6 (6)
Cl1B—C4B—C5B—N1B	−178.8 (2)	O2B—C6B—C7B—F3D	55.7 (9)
C1B—N1B—C5B—C4B	0.4 (4)	O1B—C6B—C7B—F3D	−125.6 (9)
O2A—C6A—C7A—F1C	169.3 (13)	O2B—C6B—C7B—F2B	−106.4 (8)
O1A—C6A—C7A—F1C	−8.9 (13)	O1B—C6B—C7B—F2B	72.4 (8)

### Hydrogen-bond geometry (Å, °)

**Table:**

*D*—H···*A*	*D*—H	H···*A*	*D*···*A*	*D*—H···*A*
N1A—H1NA···O1A^i^	0.94 (3)	1.79 (3)	2.727 (3)	173 (3)
N2A—H2NA···O2A^i^	0.90 (3)	1.95 (3)	2.840 (4)	175 (3)
N2A—H3NA···O1B^ii^	0.87 (3)	2.00 (2)	2.863 (3)	171 (4)
N1B—H1NB···O1B^iii^	0.87 (3)	1.87 (3)	2.734 (3)	175 (3)
N2B—H2NB···O2B^iii^	0.90 (2)	1.94 (2)	2.838 (4)	170 (2)
N2B—H3NB···O1A	0.87 (3)	1.99 (2)	2.861 (3)	175 (4)
C5A—H5AA···O2B^i^	0.97 (3)	2.29 (3)	3.210 (4)	158 (3)
C5B—H5BA···O2A^iv^	0.96 (3)	2.31 (3)	3.208 (3)	157 (3)

Symmetry codes: (i) *x*, −*y*+1, *z*−1/2; (ii) *x*, −*y*+2, *z*−1/2; (iii) *x*−1, *y*, *z*; (iv) *x*, *y*+1, *z*.

## References

[bb1] Allen, F. H., Kennard, O., Watson, D. G., Brammer, L., Orpen, A. G. & Taylor, R. (1987). *J. Chem. Soc. Perkin Trans. 2*, pp. S1–19.

[bb2] Bernstein, J., Davis, R. E., Shimoni, L. & Chang, N.-L. (1995). *Angew. Chem. Int. Ed. Engl.* **34**, 1555–1573.

[bb3] Bruker (2009). *APEX2*, *SAINT* and *SADABS* Bruker AXS Inc., Madison, Wisconsin, USA.

[bb4] Flack, H. D. (1983). *Acta Cryst.* A**39**, 876–881.

[bb5] Hemamalini, M. & Fun, H.-K. (2010*a*). *Acta Cryst.* E**66**, o557. 10.1107/S1600536810004265 PMC2983740 21580326

[bb6] Hemamalini, M. & Fun, H.-K. (2010*b*). *Acta Cryst.* E**66**, o578. 10.1107/S1600536810004447 PMC2983607 21580345

[bb7] Hemamalini, M. & Fun, H.-K. (2010*c*). *Acta Cryst.* E**66**, o464–o465. 10.1107/S1600536810002990 PMC2979927 21579876

[bb8] Jeffrey, G. A. (1997). *An Introduction to Hydrogen Bonding.* Oxford University Press.

[bb9] Jeffrey, G. A. & Saenger, W. (1991). *Hydrogen Bonding in Biological Structures.* Berlin: Springer.

[bb10] Katritzky, A. R., Rees, C. W. & Scriven, E. F. V. (1996). *Comprehensive Heterocyclic Chemistry II.* Oxford: Pergamon Press.

[bb11] Pourayoubi, M., Ghadimi, S. & Ebrahimi Valmoozi, A. A. (2007). *Acta Cryst.* E**63**, o4631. 10.1107/S1600536810002692 PMC2979840 21579865

[bb12] Pozharski, A. F., Soldatenkov, A. T. & Katritzky, A. R. (1997). *Heterocycles in Life and Society.* New York: Wiley.

[bb13] Scheiner, S. (1997). *Hydrogen Bonding. A Theoretical Perspective.* Oxford University Press.

[bb14] Sheldrick, G. M. (2008). *Acta Cryst.* A**64**, 112–122. 10.1107/S0108767307043930 18156677

[bb15] Spek, A. L. (2009). *Acta Cryst.* D**65**, 148–155. 10.1107/S090744490804362X PMC2631630 19171970

